# Diagnostic Value of Biological Parameters in Biopsy-Confirmed Thrombotic Microangiopathy–MATRIX Consortium Group

**DOI:** 10.1016/j.ekir.2025.03.019

**Published:** 2025-03-17

**Authors:** Jean-Michel Halimi, Anna Duval, Etienne Chardon, Laurent Mesnard, Marie Frimat, Fadi Fakhouri, Steven Grangé, Aude Servais, Claire Cartery, Paul Coppo, Dimitri Titeca-Beauport, Sébastien Roger, Nadine Baroukh, Nicolas Fage, Yahsou Delmas, Anne-Hélène Quérard, Guillaume Seret, Mickaël Bobot, Moglie Le Quintrec, Simon Ville, Florent von Tokarski, Sophie Chauvet, Alain Wynckel, Manon Martins, Juliet Schurder, Christelle Barbet, Bénédicte Sautenet, Philippe Gatault, Sophie Caillard, Charles Antunes, Guillaume Bayer, Carole Philipponnet, Vincent Audard, Nicolas Maillard, Vincent Vuiblet, Viviane Gnemmi, Zhour El Ouafi, Sébastien Canet, Manon Dekeyser, Éric Piver, Valentin Maisons, MATRIX Consortium Group

**Affiliations:** 1 Service de Néphrologie-Hypertension, Dialyse, Transplantation rénale, CHU de Tours, Tours, France; 2 INSERM UMR1327, ISCHEMIA, Université de Tours, Tours, France; 3 INI-CRCT, Tours, France; 4 Service de Néphrologie, CHU de Strasbourg, Strasbourg, France; 5 INSERM UMR1259, MAVIVH, Université de Tours, Tours, France; 6 Service de Néphrologie, APHP Hôpital Tenon, Paris, France; 7 INSERM, CNRS, CHU Lille, UMR9020-U1277 - CANTHER - Cancer Heterogeneity Plasticity and Resistance to Therapies, Department of Pathology, CHU Lille, Université. Lille, Lille, France; 8 Service de Néphrologie, CHU Vaudois, Lausanne, Suisse; 9 Service de Néphrologie, CHU de Rouen, Rouen, France; 10 Service de Néphrologie et Transplantation, Hôpital Universitaire Necker enfants malades, centre de référence pour les microangiopathie thrombotiques, APHP, Paris, France; 11 Service de Néphrologie, CH de Valenciennes, Valenciennes, France; 12 Service d'Hématologie, Centre de référence pour les microangiopathies thrombotiques, APHP Hôpital Saint-Antoine, Paris, France; 13 Service de Néphrologie, CHU d'Amiens, Amiens, France; 14 Service de Néphrologie, Département de médecine intensive réanimation - médecine hyperbare, CHU d'Angers, Angers, France; 15 Service de Néphrologie, CHU de Bordeaux, Bordeaux, France; 16 Centre Hospitalier Départemental de Vendée, La-Roche-Sur-Yon, France; 17 Service de Néphrologie, Pole Santé Sud Echo Le Mans, Le Mans, France; 18 Service de Néphrologie et Transplantation Rénale, Hôpital de la Conception, AP-HM, Marseille, France; 19 Aix-Marseille Université, INSERM 1263, INRAE 1260, C2VN, Marseille, France; 20 Service de Néphrologie, CHU de Montpellier, Montpellier, France; 21 Service de Néphrologie, CHU de Nantes, Nantes, France; 22 Service de Néphrologie, Hôpital Foch, Paris, France; 23 Service de Néphrologie, APHP Hôpital Européen Georges Pompidou, Paris, France; 24 Service de Néphrologie, CHU de Reims, Reims, France; 25 Service de Néphrologie, CHU de Rennes, Rennes, France; 26 Service de Néphrologie, CH de Saint-Malo, Saint-Malo, France; 27 INSERM U1246, SPHERE, Université de Tours, Université de Nantes, Tours, Nantes, France; 28 Service de Néphrologie, CHU de Brest, Brest, France; 29 Hôpital Privé Paris Essonne - Les Charmilles, Arpagon, France; 30 Service de Néphrologie, CHU de Clermont-Ferrand, Clermont-Ferrand, France; 31 Service de Néphrologie et Transplantation, Hôpitaux Universitaires Henri Mondor, APHP Université de Paris Est Créteil, INSERM U955, Institut Mondor de Recherche Biomédicale, Créteil, France; 32 Service de Néphrologie, CHU de Saint-Etienne, Saint-Etienne, France; 33 Service de Pathologie, Institut d'Intelligence Artificielle en Santé, CHU de Reims et Université de Reims Champagne Ardenne, Reims, France; 34 Service de Néphrologie, CHU de Limoges, Limoges, France; 35 Service de Néphrologie, Dialyse, soins intensifs, CH de Perpignan, Perpignan, France; 36 Service de Néphrologie, CHU Orléans, Orléans, France; 37 Laboratoire de Biochimie, Hôpital Trousseau, CHU Tours, France

**Keywords:** biological parameters, cohort, schistocytes, thrombotic microangiopathy

## Abstract

**Introduction:**

The diagnosis of thrombotic microangiopathy (TMA) relies on common biological parameters, the diagnostic value of which are unknown.

**Methods:**

The presence of common biological parameters was assessed in 967 patients with TMA from 2009 to 2023 (ClinicalTrials.gov: NCT05991245).

**Results:**

The median age was 49 (36–64) years and 53.2% were male. All TMA causes were represented (atypical hemolytic uremic syndrome [aHUS]: 41.6%, drugs: 24.9%, malignancy: 21.4%, autoimmune disease: 18.8%, infection: 7.6%, complement-mediated HUS: 6.8%, organ transplantation: 5.8%, pregnancy: 3.8%, bone marrow transplantation [BMT]: 2.9%, Shiga toxin *Escherichia coli* hemolytic uremic syndrome [STEC-HUS]: 0.6%, and thrombotic thrombocytopenic purpura [TTP]: 0.6%). The presence of TMA-related parameters concerned virtually all patients with TTP but varied widely for the other patients as follows: anemia: 81.7%, high lactate dehydrogenase (LDH) (75.4%), low haptoglobin (53.7%), and thrombocytopenia (40.3%). Their diagnostic performance was accurate only for TTP. Eleven distinct ways were used for schistocyte metrics and reporting. Relying on schistocyte presence as the single diagnostic criterion would lead to missed diagnosis in 23.8% (STEC-HUS) to 86.4% (BMT) of patients (for anemia: 8.2%–22.3%; thrombocytopenia: 31.8%–67.9%; high LDH: 10.0%–40.7%, low haptoglobin: 0%–70.4%, according to the causes of TMA). The overall risk of missed diagnosis using these parameters was ≥ 50% in all TMA, except in TTP. The best diagnostic performances were obtained when fibrinogen levels were < 5 g/l, creatinine ≥ 300 μmol/l, prothrombin time (PT) < 90%; and when TMA causes were TTP, STEC-HUS, infection, or complement-mediated HUS.

**Conclusion:**

Common biological parameters miss the diagnosis in more than 50% of TMA except when fibrinogen is < 5 g/l, creatinine ≥ 300 μmol/l, and PT < 90%. Schistocyte reporting is heterogenous, and its results are usually deceptive in TMA.

## Graphical abstract

**Figure:**
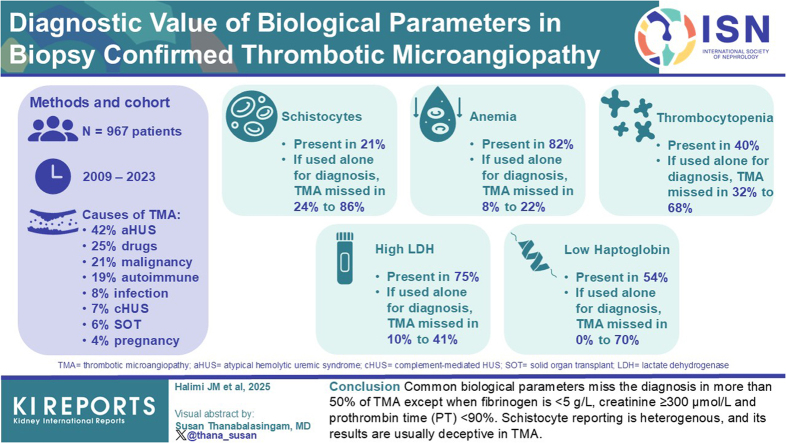

See Commentary on Page 1625

First described in 1952,^1^ TMAs present with a pathologic alteration of the microvasculature, characterized by disseminated microthrombi occluding both arterioles and capillaries.^2^^,^^3^ TMAs are suspected in patients with microangiopathic hemolytic anemia, low platelet counts, and organ injuries.^3^

Since 2002, “thrombocytopenia, schistocytes, extremely elevated serum levels of LDH” have been considered diagnostic TMA hallmarks.^4^ The guidelines of the International Council for Standardization in Hematology^5^ include requesting a schistocyte count for suspected TMA diagnosis. They also state that “a robust morphological threshold for suspected TMA diagnosis is set at the percentage of schistocytes above 1%” and that “evidence-based reference values of the schistocyte percentage using optical microscopy on peripheral blood smears are 1% or less in normal adults.”^5^ Nevertheless, the absence of schistocytes does not rule out the diagnosis of TMA.^3^^,^^4^

Although these biological parameters have been used for decades as a means to suspect the diagnosis of TMA, their diagnostic value is unclear because biopsies are performed only in a limited number of cases, leading to selection bias. To ensure proper analysis of the diagnostic values of these biological parameters and identify patients in whom these biological parameters have the diagnostic performance, it is crucial to analyze large cohorts of patients with TMA proven by biopsy. However, such analyses are presently lacking.

In the present study, we assessed the diagnostic value of usual biological parameters (schistocytes, LDH, haptoglobin, anemia, and thrombocytopenia) for the diagnostic of TMA in 967 unselected patients who had a TMA from all causes proven by renal biopsy and biological results at the time of biopsy from 25 French hospital centers.

## Methods

### Study Design and Patient Selection

This longitudinal study was conducted in 25 French hospital centers, 14 of them (56.0%) affiliated to the French National Reference Center of TMA (www.cnr-mat.fr) (Supplementary Figure S1). Medical records of all patients were obtained to collect relevant information. Patients admitted to French hospitals between January 1, 2009 and December 31, 2023 with the following selection criteria were included: (i) aged ≥ 18 years; and (ii) biopsy of native kidney, performed because of elevated serum creatinine, showing TMA regardless of its cause. Kidney biopsies were performed because of acute kidney injury and/or proteinuria, in the absence of hematological evidence of TMA.

### Data Sharing Statement and Ethical Approval Information

Ethical approval has been obtained from the Centre Val de Loire Region ethical platform (n°2022-59). Procedures for data collection and management were approved by the Commission Nationale de l'Informatique et des Libertés, the French National Commission for Data Protection and Liberties protecting human rights in France. The database was approved by Commission Nationale de l'Informatique et des Libertés (registration number F20221110095846). Information about data anonymous use was provided, but written consent was not required by French law. The protocol is registered on ClinicalTrials.gov (NCT05991245). Access to anonymized data may be requested within reasonable limits.

### Data Collected

Demographic data and TMA biological features were reported by the physician of each center (Supplementary Table S1)^6^ at presentation. All diagnostic tests were performed in local centers. Serum creatinine, hemoglobin, platelet count, fibrinogen, PT, haptoglobin, and LDH were measured using usual methods. However, the upper normal limit of LDH and lower limit of detection of haptoglobin could be different from one laboratory to another, as expected in a real-life study.

We carefully analyzed the metrics and threshold reporting value of schistocytes. We defined the sensitivity as the proportion of patients with a given parameter to suspect renal TMA, and we assessed this sensitivity according to creatinine levels, fibrinogen, PT, and TMA causes. Native kidney biopsies were analyzed and TMA was diagnosed by local renal pathologists according to usual criteria.^7^ TMA causes were defined by local clinicians, then adjudicated by 2 authors (J-MH and VM) and classified according to the Kidney Disease: Improving Global Outcomes (KDIGO) classification.^7^, ^8^, ^9^ Because patients were included during a long period from 2009 to 2023 period, many of them did not have a genetic testing, and in some patients with identified causes of TMA, the screening for complement gene rare variant was not considered warranted by investigators. Overall, 390 patients had genetic screening.

aHUS was defined as the absence of the above-mentioned KDIGO causes (Supplementary Table S1). As indicated in Supplementary Table S1, the 2017 KDIGO classification of TMA was used. In this classification, hypertensive emergency was not present as a cause of TMA. Many of our patients, including those with aHUS, could have severe or malignant hypertension. However, initial blood pressure values and/or fundoscopy results were not available.

### Statistical Analyses

Data are presented as median (interquartile range) for quantitative variables and counts and percentages for categorical variables. Comparisons were made using Wilcoxon rank test, chi-square, and Fisher exact test, as appropriate. Multivariable logistic regressions were performed to assess the associations between the presence of hemolysis parameters and TMA, according to clinical and biological parameters. Statistical analyses were performed using SAS 9.4 (SAS Institute Inc., Cary, NC).

## Results

### Baseline Characteristics

Overall, 967 patients with TMA proven by renal biopsy were included. The median age was 49 (36–64) years and 53.2% of patients were male. Most patients had available data regarding hemoglobin, platelet count, haptoglobin, LDH levels, and schistocytes. All KDIGO causes of TMA were represented, including aHUS (41.6%) (including complement-mediated aHUS [6.8%]), drugs (24.9%), malignancy (21.4%), autoimmune disease (18.8%), infection (6.8%). Other KDIGO causes were much less frequent (Table 1).

**Table 1:** Characteristics and data availability in patients with TMA

Characteristics	Number of patients with available data	Parameters
Demographics		
Age (yrs)	967 (100%)	49 (36–64)
Male sex (%)	967 (100%)	53.2
Availability of biological parameters		
Hemoglobin (g/l)	964 (99.7%)	101 (85–118)
Platelet count (/μl)	961 (99.4%)	180 (107–252)
Low haptoglobin (%)	853 (88.2%)	53.7
High LDH (xULN)	842 (87.1%)	1.80 (1.04–2.90)
Schistocyte > 1%	761 (78.7%)	34.0%
Fibrinogen levels (g/l)	621 (64.2%)	4.40 (3.50–5.30)
Serum creatinine (μmol/l)	963 (99.6%)	258 (151–500)
Prothrombin time (%)	895 (92.6%)	97 (86–100)
TMA-associated conditions		
Drugs (%)	967 (100%)	24.9
Malignancy (%)	967 (100%)	21.4
Autoimmune (%)	967 (100%)	18.8
Atypical HUS (%)	967 (100%)	41.6
Complement-mediated aHUS (%)	967 (100%)	6.8
Infection (%)	967 (100%)	7.6
Organ transplantation (%)	967 (100%)	5.8
Pregnancy (%)	967 (100%)	3.8
BMT (%)	967 (100%)	2.9
STEC-HUS (%)	967 (100%)	0.6
MMACHC (%)	967 (100%)	1.1
TTP (%)	967 (100%)	0.6

BMT, bone marrow transplantation; HUS, hemolytic uremic syndrome; LDH, lactate dehydrogenase; MMACHC, methylmalonic aciduria and homocystinuria type C protein gene variant; STEC-HUS, Shiga toxin *Escherichia coli*–associated HUS; TMA, thrombotic microangiopathy; TTP, thrombotic thrombocytopenic purpura.

Some patients had > 1 cause of TMA. Results in % or median (interquartile range).

### Laboratory Reporting of Schistocytes

The reporting of schistocytes’ results was investigated for each laboratory. Eleven different ways were used to report schistocytes (Table 2). Overall, 13 laboratories used quantitative results, 4 used semiquantitative results, 7 used a mix of quantitative or semiquantitative results, and 1 used a binary reporting (yes vs. no) (Table 2). Among laboratories expressing results as quantitative results, thresholds for the presence of schistocytes ranked as follows: 0.1% (1 laboratory), 0.25% (1 laboratory), 0.5% (1 laboratory), and 1% (8 laboratories). Eight laboratories used adjective-based reporting, and among them, the same adjective did not correspond to the same quantitative value (for instance, “rare schistocytes” corresponded to 1–2 per field in one laboratory, 0.3–0.9% in another laboratory, and no specific threshold for a different laboratory; “numerous schistocytes” corresponded to 6–10 schistocytes per field in one laboratory, 5.0%–9.9% in another laboratory, or no specific threshold in a different laboratory) (Table 2).

**Table 2:** Laboratory schistocyte reporting among the 25 centers

Number of centers	Number of patients	Type of result	Schistocyte reporting / threshold value	Schistocyte > 1%
4	179	quantitative	0.1%	>1% = yes
1	38	semiquantitative / quantitative	< 0.5%	no
			quantitative when ≥ 0.5%	0.5%–1% = no; >1% = yes
1	48	semiquantitative	rare: 1–2 per field, 50× magnification	no
			some: 3–5 per field, 50× magnification	no
			numerous: 6–10 per field, 50× magnification	no
			very numerous: >10 per field, 50× magnification	yes
1	15	quantitative	> 0.25%	0.25%–1% = no; >1% = yes
1	19	semiquantitative	> 0.5%, variable range	0.5%–1% = no; >1% = yes
1	18	quantitative	> 0.5%	0.5%–1% = no; >1% = yes
1	58	semiquantitative	rare: 0.3%–0.9%	no
			some: 1.0%–1.9%	yes
			relatively numerous: 2.0%–4.9%	yes
			numerous: 5.0%–9.9%	yes
			very numerous: ≥10%	yes
1	19	semiquantitative	rare	no
			present	yes
			numerous	yes
5	270	semiquantitative/quantitative	rare or some	no
			≥1%	yes
7	206	quantitative	≥1%	yes
1	93	binary	≥1%	yes
1	4	NA: no patient with data	NA	NA

NA: not applicable.

### Diagnostic Value of all Biological Parameters

**Figure 1:**
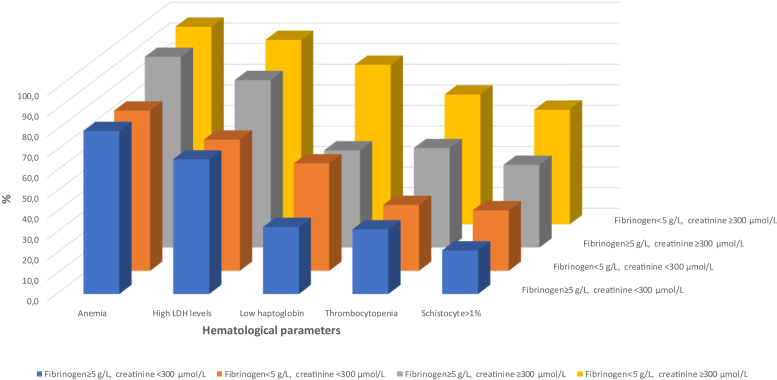
Impacts of fibrinogen levels and prothrombin time on the diagnostic value of biological parameters to suspect TMA. The proportion of patients with a given parameter in our patients with TMA was expressed as the percentage of patients with the biological parameters, according to fibrinogen levels and prothrombin time. LDH, lactate dehydrogenase; TMA, thrombotic microangiopathy.

Biological parameters’ presence at the time of TMA diagnosis ranked as follows: anemia (81.7%), high LDH (75.4%), low haptoglobin (53.7%), thrombocytopenia (40.3%), and schistocytes > 1% (20.5%) (Figure 1). The diagnostic value of schistocytes > 1% was greater in patients with thrombocytopenia than in those without (55.4% vs. 16.4%, *P* < 0.0001).

### Impact on the Risk of Missed Diagnosis of TMA According to Their Cause

Usual biological parameters used to suspect TMA are almost of 100% sensitivity for the 6 patients with TPP (0.6% of the population) (Table 3).

**Table 3:** Rate of missed diagnosis when using one single biological parameter or all combined according to the cause of TMA

Cause of TMA	Schistocytes >1%	Anemia	Thrombocytopenia	High LDH	Low haptoglobin	Anemia, thrombocytopenia and high LDH	All 5 parameters combined
TTP	0	0	0	0	16.7	0	75.0^a^
STEC-HUS	23.8	13.6	31.8	14.3	19.1	42.9	57.1
Infection	47.6	8.2	46.6	18.5	29.9	46.2	58.7
C-mediated TMA	49.2	12.1	48.5	10.0	21.9	46.7	63.3
Pregnancy	56.7	21.6	48.7	22.6	42.4	48.4	73.3
Organ transplantation	55.8	7.1	46.3	16.3	29.2	49.0	76.1
MMACHC	66.7	18.2	54.6	10.0	0	50.0	80.0
Autoimmune	60.7	10.4	54.4	23.8	46.5	58.9	77.9
Drugs	68.9	17.8	57.3	21.8	45.4	62.4	79.6
aHUS	68.6	22.3	65.7	25.7	47.9	65.4	79.4
Malignancy	72.0	19.3	59.7	28.4	50.6	66.5	80.7
BMT	86.4	14.3	67.9	40.7	70.4	85.2	96.2

aHUS, atypical hemolytic uremic syndrome; BMT, bone marrow transplantation; LDH, lactate dehydrogenase; MMACHC, methylmalonic aciduria and homocystinuria type C protein gene variant; STEC-HUS, Shiga toxin *Escherichia coli* hemolytic uremic syndrome; TMA, thrombotic microangiopathy; TTP, thrombotic thrombocytopenic purpura.

a 2 missing values among 6 patients with TTP.

In marked contrast, these biological parameters were often absent in the remaining 99.4% of the population. The risk of missed TMA diagnosis was ≥ 50% but varied widely according to the cause of TMA; relying only on the presence of schistocytes as the single diagnostic criterion would lead to miss the diagnosis of TMA in 23.8% (STEC-HUS) to 86.4% (BMT) of patients (Table 3). The respective values for other biological parameters were as follows: anemia: 8.2% (infection) to 22.3% (aHUS), thrombocytopenia: 31.8% (STEC-HUS) to 67.9% (BMT), high LDH: 10.0% (methylmalonic aciduria and homocystinuria type C protein gene variant) to 40.7% (BMT), low haptoglobin: 0% (methylmalonic aciduria and homocystinuria type C protein gene variant) to 70.4% (BMT) (Table 3).

We then assessed the impact of the presence of anemia, high LDH, and thrombocytopenia combined on the risk of missed diagnosis in all patients (except those with TTP): it ranked from 42.9% (STEC-HUS) to 85.2% (BMT) (Table 3). When all these 5 biological parameters combined were considered, the risk of missed diagnosis ranked from 57.1% (STEC-HUS) to 96.2%) (Table 3).

### Impact of Clinical and Biological Parameters and Implication for Clinical Practice

**Figure 2:**
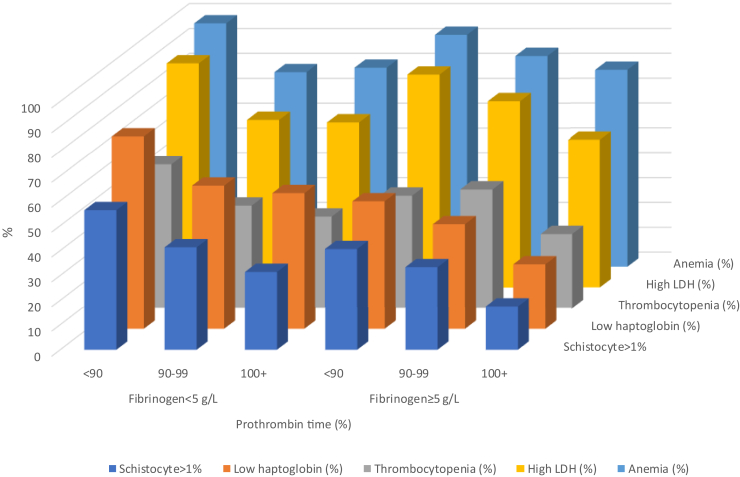
Combined impact of fibrinogen and serum creatinine levels. The proportion of patients with a given parameter in our patients with TMA was expressed as the percentage of patients with the biological parameters, according to fibrinogen and serum creatinine levels. LDH, lactate dehydrogenase; TMA, thrombotic microangiopathy.

As shown in Table 4, the diagnostic value of anemia, high LDH, low haptoglobin, and thrombocytopenia was not influenced by sex and age categories. In contrast, the diagnostic value of these 5 parameters was statistically modified by serum creatinine and PT **(**Table 4**)**. In addition, schistocytes (30.5% vs. 42.0%) and low haptoglobin (64.0% vs. 40.1%) were more frequently observed in patients with serum fibrinogen < 5 g/l versus other patients (Table 4). These findings were confirmed in multivariable analyses (Table 4). The impacts of fibrinogen and creatinine combined, and fibrinogen and PT combined on the sensitivity of the biological parameters are depicted in Figure 2a and b.

**Table 4:** Impact of clinical and biological parameters on the diagnostic value of biological parameters: multivariable analyses

Parameters	Schistocytes >1% (%)			Anemia (%)			Thrombocytopenia (%)			High LDH (%)			Low haptoglobin		
	%	aOR (95%)	*P* value	%	aOR (95%)	*P* value	%	aOR (95%)	*P* value	%	aOR (95%)	*P* value	%	aOR (95%)	*P* value
Women vs. men	37.1 vs. 31	1.32 (0.097–1.78)	0.073	84.7 vs 79.1	1.47 (1.05–2.05)	0.024	43.4 vs 37.5	1.28 (0.98–1.65)	0.066	78.4 vs 72.8	1.30 (0.99–1.87)	0.058	56.6 vs 51.0	1.25 (0.96–1.64)	0.103
Age < 65 vs. ≥ 65 yrs	35.1 vs. 30.6	1.23 (0.86–1.76)	0.260	83.8 vs 81.1	1.21 (0.82–1.80)	0.342	41.0 vs 38.0	1.13 (0.84–1.53)	0.423	77.4 vs 69.4	1.51 (1.06–2.14)	0.022	54.6 vs 50.7	1.17 (0.85–1.60)	0.330
Creatinine ≥ 300 vs. < 300, μmol/l	46.3 vs. 23.1	2.87 (2.09–3.91)	<0.001	94.4 vs 72.5	6.37 (4.02–10.1)	<0.001	55.3 vs 29.5	2.96 (2.26–3.88)	<0.001	84.9 vs 67.3	2.74 (1.95–3.85)	<0.001	66.8 vs 42.5	2.72 (2.05–3.60)	<0.001
Fibrinogen < 5 vs. ≥5 g/l	42.0 vs. 30.5	1.65 (1.11–2.44)	0.013	85.5 vs 85.9	0.97 (0.60–1.57)	0.890	45.1 vs 39.7	1.25 (0.89–1.76)	0.203	75.8 vs 73.7	1.12 (0.74–1.68)	0.602	64.0 vs 40.1	2.66 (1.85–3.81)	<0.001
PT ≥ 90% vs. < 90%	44.2 vs. 27.9	2.05 (1.48–2.82)	<0.001	92.7 vs 76.3	3.97 (2.47–6.37)	<0.001	51.2 vs 33.8	2.05 (1.54–2.72)	<0.001	87.1 vs 68.5	3.09 (2.08–4.60)	<0.001	64.1 vs 46.7	2.03 (1.51–2.74)	<0.001

aOR, adjusted odds ratio; LDH, lactate dehydrogenase; PT, prothrombin time.

The rate of the biological parameters of TMA increased with increasing serum creatinine (Supplementary Figure S2A) and decreased with increasing PT (Supplementary Figure S2B). However, only the rate of low haptoglobin and schistocytes decreased in patients with fibrinogen levels ≥ 5 g/l versus the other 2 groups of patients (Supplementary Figure S2C).

Patients with the best diagnostic performances for these parameters were those with both fibrinogen levels < 5 g/l and serum creatinine ≥ 300 μmol/l (29% of the population) (Supplementary Table S2). Patients with both fibrinogen levels ≥ 5 g/l and serum creatinine < 300 μmol/l had the highest risk of missed diagnosis (16% of the population) (Supplementary Table S2).

## Discussion

In the present study, in 967 patients with proven TMA, the reporting of schistocytes was highly heterogenous among laboratories. The biological parameters commonly used to suspect TMA, especially schistocytes, were present only in a minority of patients. These parameters diagnostic value was excellent for patients with TTP but low in all other causes of TMA. Relying solely on the result of these biological parameters would lead to missing the diagnosis of TMA in ≥ 50% of causes, and even more in patients with both fibrinogen levels ≥ 5 g/l, low PT and/or serum creatinine < 300 μmol/l.

Overall, the biological parameters we studied *we*re absent in many patients with TMA; this is especially for schistocytes, which reporting, and metrics varied widely among laboratories. Only 15% to 57% of patients, according to the cause of TMA, had anemia, high LDH, and thrombocytopenia combined (4%–43%, when all of these 5 biological parameters combined were considered). The presence of schistocytes was observed in only 20.5% of patients. The International Council for Standardization in Hematology recently recommended that the percentage of schistocytes suggestive of microangiopathic hemolytic anemia should be > 1%.^3^ In another report, based on patients with proven TMA, the prevalence of schistocytes was 42 of 97 (43%).^10^ Altogether and with other findings,^3^^,^^4^^,^^11^ these results strongly support the following views: that (i) renal TMA should be considered in patients with unexplained renal failure, and the diagnosis work-up should systematically include markers of hemolysis; (ii) the request of a schistocyte count should be repeated several times in patients with renal dysfunction because schistocytes may be initially absent^3^^,^^4^ and increase with time^11^; and (iii) there is a strong clinical need for standardization of schistocyte quantification and reporting.^12^^,^^13^ In the near future, imaging flow cytometry and machine learning may improve sensitivity and reproducibility of schistocytes identification.^14^^,^^15^

The diagnostic value of these parameters was excellent for patients with TTP (0.6% of all patients), and at least in these patients, relying on these parameters is appropriate and corresponds to clinical practice. In marked contrast, in the remaining 99.4% of patients with proven TMA, the interpretation of the results of these parameters was difficult and often deceitful, especially when TMA is due to autoimmune diseases, drugs, aHUS, malignancy, and BMT. A case series of 35 patients with TMA without evidence of systemic hemolysis was reported in 2018: none of them had TTP and most causes of TMA were drugs or autoimmune diseases.^16^ Drugs include bevacizumab, a VEGF-trap or tyrosine kinase inhibitor,^17^ even used as intravitreal injections.^18^ Other case reports of patients with TMA and little or no evidence of hemolysis were reported in antineutrophil cytoplasmic autoantibody vasculitis,^19^ lupus nephritis,^20^ and IgA nephropathy.^21^

In our study, common biological parameters missed the diagnosis in over 50% of TMA cases. However, this figure may be overestimated due to our selection criteria and the availability of only 1 biological evaluation. These results must be replicated in other studies.

We found that the results of the biological parameters should be interpreted according to renal function (serum creatinine ≥ 300 vs. < 300 μmol/l), fibrinogen levels (< 5 vs. ≥ 5 g/l) and PT (< 90% vs. ≥ 90%). Markers of hemolysis were more frequently observed in patients with severe renal dysfunction. Interestingly, it was recently demonstrated that heme may aggravate TMA by local complement activation leading to further renal parenchymal damage.^22^^,^^23^ The normality of several markers of hemolysis was deceptive in patients with high fibrinogen levels.

The appropriate use of fibrinogen levels and PT may help clinicians when the diagnosis of TMA is uncertain. For example, haptoglobin normal levels in patients with high fibrinogen levels do not rule out the diagnosis of TMA because this normal value can result from decreased free haptoglobin due to hemolysis combined to increased synthesis secondary to inflammation. Similarly, it should be interesting to assess and understand the exact relationship between haptoglobin and fibrinogen, and between PT and TMA biological parameters. It is acknowledged that low haptoglobin had an excellent sensitivity and specificity for the diagnosis of hemolysis,^24^^,^^25^ and it was stated that the diagnostic value of low haptoglobin is not affected by inflammation in TMA.^25^ In marked contrast, our results clearly indicate that low haptoglobin is much less frequently observed in patients with high fibrinogen levels in patients with TMA. As stated by others, “given the widespread use of haptoglobin testing, it is vital that clinicians and laboratory staff understand the principles and correct interpretation of this test.”^26^ The inverse relationship between the presence of hemolysis parameters and PT levels was observed in the absence of disseminated intravascular coagulation, because PT was usually in the normal range and was independent of fibrinogen levels. Interestingly, in a recent study,^27^ a relationship was observed between international normalized ratio > 1.3 and causes of TMA. From a clinical perspective, our findings could impact clinical practice. Diagnostic work-up for hemolysis should systematically include PT, fibrinogen, and serum creatinine; conversely, diagnosis work-up for acute kidney injury should systematically incorporate hemolysis parameters, PT, and fibrinogen. These novel findings are decisive for clinical practice. Our data indicate that the risk of missing the diagnosis of TMA is very elevated among patients with acute kidney injury of unclear origin. This is especially true for those with both fibrinogen levels ≥ 5 g/l and serum creatinine < 300 μmol/l (16% of the population). The diagnostic value of biological parameters remains acceptable in patients with fibrinogen < 5 g/l and serum creatinine ≥ 300 μmol/l (29% of the population).

Our study has several strengths. First, it is likely the largest worldwide study of patients with biopsy-proven renal TMA. Second, most patients had a complete TMA diagnostic work-up. Third, the diagnostic value of schistocytes presence, quantification, and reporting was analyzed in detail. Fourth, we provided new data regarding the interpretation of TMA-related biological parameters according to fibrinogen, creatinine, and PT. Fifth, we identified subgroups of patients in whom diagnosis of TMA would be easily missed and those in whom the diagnostic value of common laboratory parameters remained acceptable. This information could help physicians appreciate the need for kidney biopsy to detect TMA and balance it with risk of bleeding after kidney biopsy.^28^^,^^29^

As stated in the Methods section, the 2017 KDIGO classification of TMA was used, and patients could have hypertensive emergency; whether hypertension could be the cause or trigger of TMA is presently unclear.

Our study has also some limitations. First, the results of only 1 blood sample was available at the time of TMA diagnosis, preventing assessment of the kinetics of biological parameters over time. However, clinicians do not always repeat schistocyte and haptoglobin requests in patients when the initial evaluation yields normal results. Second, the specificity of the biological parameters could not be assessed and schistocytes may be found in other situations.^30^, ^31^, ^32^, ^33^ Third, only patients who were biopsied were included in this study, biopsied patients may have different clinical and biological profiles than other patients with TMA with no histological proof of TMA. Methods of measurement of biological parameters were not standardized across the centers, and it is possible that some were automated assessed whereas others were manually assessed. The experience of technicians may also vary across laboratories and within a given laboratory.

Not all patients with suspicion of TMA have a kidney biopsy to confirm TMA in usual practice, introducing a selection bias for the diagnosis of renal TMA. In the present study, we approached the problem from another perspective: we analyzed 967 patients with biopsy-proven TMA from the French MATRIX consortium because it is the only way to assess the sensitivity of the biological parameters for the diagnosis of renal TMA. This study design does not represent this usual practice but is adapted for the purpose of our study.

In conclusion, our results indicate that schistocyte reporting is heterogenous and biological parameters associated with TMA including schistocytes are deceptive in TMA in most patients. Our findings can be easily used in clinical practice to improve the diagnosis of TMA. They imply that fibrinogen levels and PT should systematically be part of the diagnosis work-up in patients with acute kidney injury even when TMA is not formerly suspected, to avoid missing patients with TMA when there are few or no evidence of microangiopathic hemolytic anemia.

## Appendix

### List of the members of the MATRIX Consortium Group

Charles Antunes, Vincent Audard, Nadine Baroukh, Mickaël Bobot, Isabelle Brocheriou, David Buob, Sophie Caillard, Sébastien Canet, Claire Cartery, Sophie Chauvet, Manon Dekeyser, Yahsou Delmas, Jean-Paul Duong, Anna Duval, Zhour El Ouafi, Nicolas Fage, Fadi Fakhouri, Sophie Ferlicot, Véronique Frémeaux-Bacchi, Marie Frimat, Viviane Gnemmi, Jean-Michel Goujon, Steven Grangé, Jean-Michel Halimi, Nicolas Kozakowski, Moglie Le Quintrec, Nicolas Maillard, Valentin Maisons, Manon Martins, Ingrid Masson, Laurent Mesnard, Charlotte Mussini, Jérome Olagne, Hélène Perrochia, Carole Philipponnet, Anne-Hélène Quérard, Marion Rabant, Nolwenn Rabot, Guillaume Seret, Aude Servais, Juliet Schurder, Elodie Standley, Dimitri Titeca-Beauport, Simon Ville, Florent von Tokarski, Vincent Vuiblet, Alain Wynckel.

## Disclosure

J-MH reported honoraria for lectures and travel grant from Alexion and AstraZeneca. SCai reported honoraria for lectures and travel grant from Alexion and Samsung Bioepis. MF reported speaker bureau fees from Alexion, AstraZeneca, and Novartis. FF reported receiving honoraria for lectures from Alexion, AstraZeneca, Sobi, Apellis, and Novartis. SG reported travel grants and honoraria for lectures from Alexion, AstraZeneca, and Lilly. PC reported honoraria for lectures from Takeda, Sanofi, Alexion, and Sobi. DT-B reported honoraria for lectures and travel grant from CSL Vifor. YD reported honoraria for educational events from, support for attending meeting from, and advisory board membership with Novartis, Sanofi, and Samsung. SCha reported honoraria for lectures, support for attending meeting from Novartis, Sobi, and CSL Vifor. VA reported support for attending meeting and advisory board from Sanofi Genzyme, AstraZeneca, Alnylam, Vifor Pharma, and Bayer. All the other authors declared no competing interests.

## Supplementary Material

Supplementary File (PDF) **Figure S1.** Distribution of the 25 MATRIX investigator centers in France. These centers are located throughout metropolitan France. The centers are indicated by a colored circle: red for the coordinating center and green for the other recruiting centers. **Figure S2.** (A) when patients were separated according to serum creatinine levels (< 200 μmol/l, 200–299 μmol/l and ≥ 300 μmol/l), the rate of all biological parameters of TMA increased with increasing serum creatinine groups from < 200 μmol/l to ≥ 300 μmol/l. (B) When the patients were separated according to prothrombin time (< 90%, 90%–99%, ≥ 100%), the rate of all biological parameters of TMA progressively decreased from < 90% to ≥ 100% prothrombin time groups. (C) When the patients were stratified according to serum fibrinogen levels (< 4 g/l, 4–4.99 g/l, and ≥ 5 g/l), the rate of low haptoglobin and schistocytes (but not the rate of anemia, high LDH, or thrombocytopenia) decreased in patients with fibrinogen levels ≥ 5 g/l versus the other 2 groups of patients. **Table S1.** Definitions. **Table S2.** Impact of fibrinogen and serum creatinine on sensitivity of biological parameters to suspect renal TMA.

## Footnotes

Supplementary File (PDF)
**Figure S1.** Distribution of the 25 MATRIX investigator centers in France. These centers are located throughout metropolitan France. The centers are indicated by a colored circle: red for the coordinating center and green for the other recruiting centers. **Figure S2.** (A) when patients were separated according to serum creatinine levels (< 200 μmol/l, 200–299 μmol/l and ≥ 300 μmol/l), the rate of all biological parameters of TMA increased with increasing serum creatinine groups from < 200 μmol/l to ≥ 300 μmol/l. (B) When the patients were separated according to prothrombin time (< 90%, 90%–99%, ≥ 100%), the rate of all biological parameters of TMA progressively decreased from < 90% to ≥ 100% prothrombin time groups. (C) When the patients were stratified according to serum fibrinogen levels (< 4 g/l, 4–4.99 g/l, and ≥ 5 g/l), the rate of low haptoglobin and schistocytes (but not the rate of anemia, high LDH, or thrombocytopenia) decreased in patients with fibrinogen levels ≥ 5 g/l versus the other 2 groups of patients. **Table S1.** Definitions. **Table S2.** Impact of fibrinogen and serum creatinine on sensitivity of biological parameters to suspect renal TMA.
