# Supplementary material for: Diagnostic Value of Biological Parameters in Biopsy-Confirmed Thrombotic Microangiopathy–MATRIX Consortium Group
Source: Kidney Int Rep. 2025 Mar 17;10(6):1950–9. doi: 10.1016/j.ekir.2025.03.019 (PMC12232970; doi:10.1016/j.ekir.2025.03.019)

Supplemental Materials

Supplemental Table 1: Definitions

	Definitions
Anemia	Hemoglobin <120 g/L in women, <130 g/L in men
Thrombocytopenia	platelet count <150,000/µL
Low haptoglobin	<lower normal limit of normal for the laboratory (usually <0.25 or <0.30 g/L)
High LDH	>upper normal limit of the laboratory
Schistocyte>1%	Presence of schistocyte>1%
Schistocyte≥0.1%	Presence of schistocyte, regardless of the number

**Causes of TMA**	**Definitions**
**TTP**	<10% ADAMTS13 activity.
**Cobalamin-deficiency**	Pathogenic variants in the MMACHC gene were present (*Ref.10*).
**STEC**	Shigatoxin due E.Coli associated with TMA (immunological method or PCR).
**BMT**	Renal TMA in a patient with BMT.
**Pregnancy**	Renal TMA in a pregnant woman (or during the post-partum period).
**Infection**	Renal TMA in a patient with any active infection (except STEC-HUS at the time of diagnosis).
**Malignancy**	Renal TMA in patients with active solid cancer or hematological malignancy at the time of diagnosis.
**Transplantation**	Renal TMA in a patient with solid organ transplantation (any solid organ transplantation except kidney transplantation)
**Auto-immune**	Renal TMA in a patient with auto-immune diseases including lupus, scleroderma crisis, Sjogren disease, anti-phospholipid syndrome, cryoglobulinemia.
**Drug-induced**	Renal TMA associated with the use of drugs including anti-VEGF drugs, calcineurin inhibitors, gemcitabine, mTOR inhibitors (*Ref. 9*).
**Atypical HUS (aHUS)**	Defined as the absence of the above-mentioned KDIGO TMA causes.
**Complement-mediated aHUS**	Among patients with aHUS, presence of alternative pathway activation (auto-antibodies or pathogenic variants)

Abbreviations: BMT: Bone Marrow Transplantation; HUS: Hemolytic and Uremic Syndrome; KDIGO: Kidney Disease Improving Global Outcomes;

STEC: Shiga-like Toxin-producing E. coli; TMA, Thrombotic microangiopathy; TTP: Thrombotic Thrombocytopenic Purpura

Supplemental Table 2: Impact of fibrinogen and serum creatinine on sensitivity of biological parameters to suspect renal TMA

	Fibrinogen ≥5 g/L creatinine <300 µmol/L	Fibrinogen <5 g/L creatinine <300 µmol/L	Fibrinogen ≥5 g/L creatinine ≥300 µmol/L	Fibrinogen <5 g/L creatinine ≥300 µmol/L	p value
**Proportion of the population**	**16%**	**39%**	**16%**	**29%**	
**Parameter**					
Anemia	79.2	77.8	92.1	96.0	<0.0001
High LDH	65.1	64.8	80.7	90.1	<0.0001
Low haptoglobin	31.9	54.2	47.7	77.8	<0.0001
Thrombocytopenia	31.7	32.6	50.0	64.0	<0.0001
Schistocyte>1%	21.2	29.4	40.2	55.8	<0.0001

Sensitivity defined as the proportion of patients with a given parameter among patients with biopsy-proven renal TMA

Supplemental Figure 1: Distribution of the 25 MATRIX investigator centers in France

The centers are indicated by a colored circle: red for the coordinating center and green for the other recruiting centers. these centers are located throughout metropolitan France.

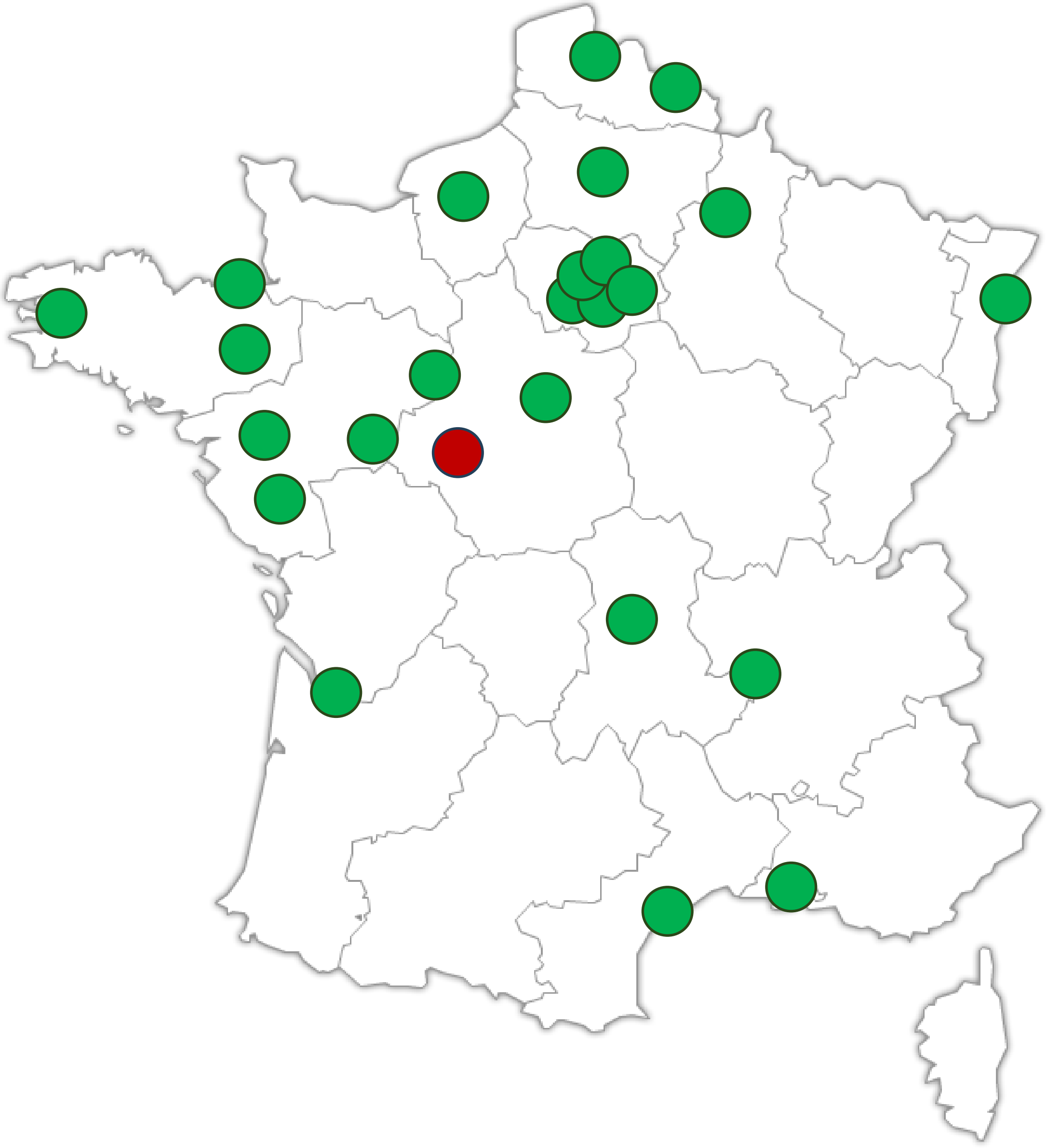

Supplemental Figure 2a: *when patients were separated according to serum creatinine levels (<200 µmol/L, 200-299 µmol/L and ≥300 µmol/L), the rate of all biological parameters of TMA increased with increasing serum creatinine groups from <200 µmol/L to ≥300 µmol/L.*

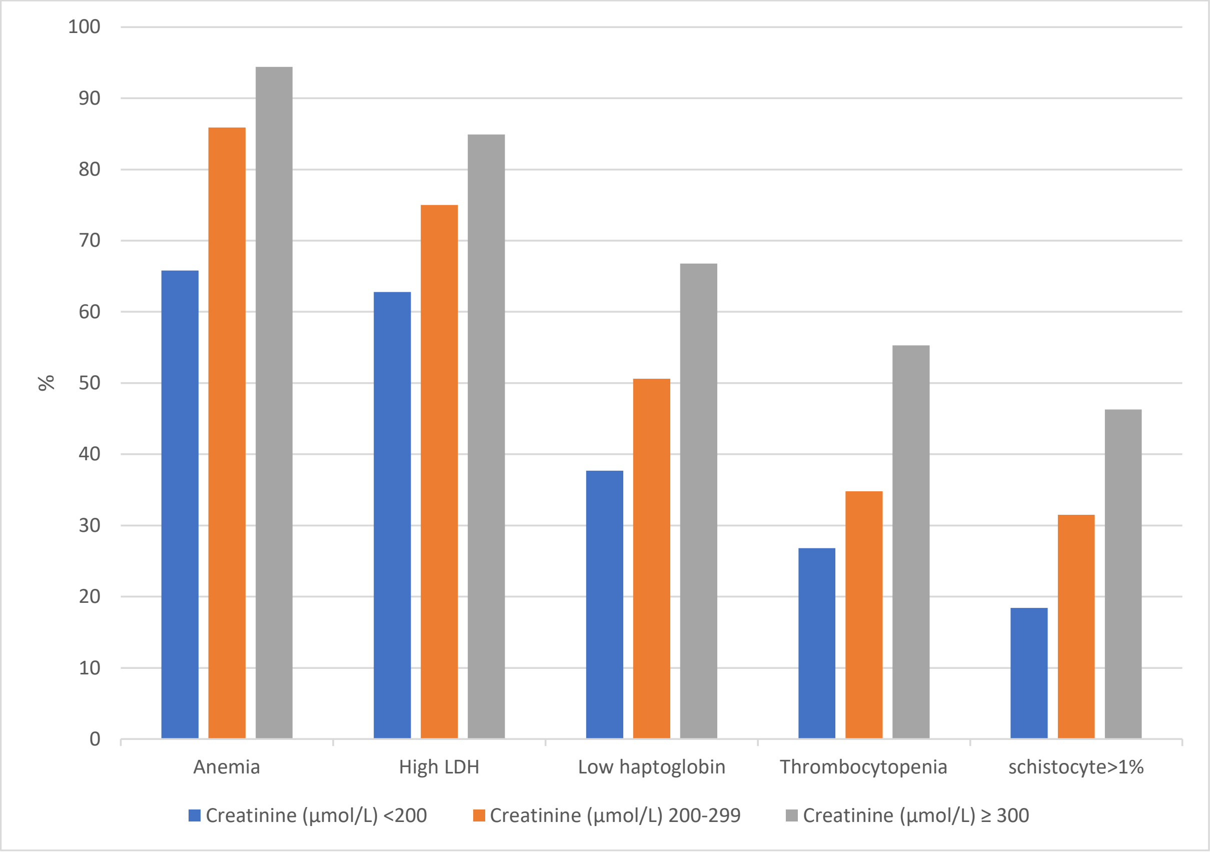

Supplemental Figure 2b: *when the patients were separated according to prothrombin time (<90%, 90-99%, ≥100%), the rate of all biological parameters of TMA progressively decreased from <90% to ≥100% prothrombin time groups.*

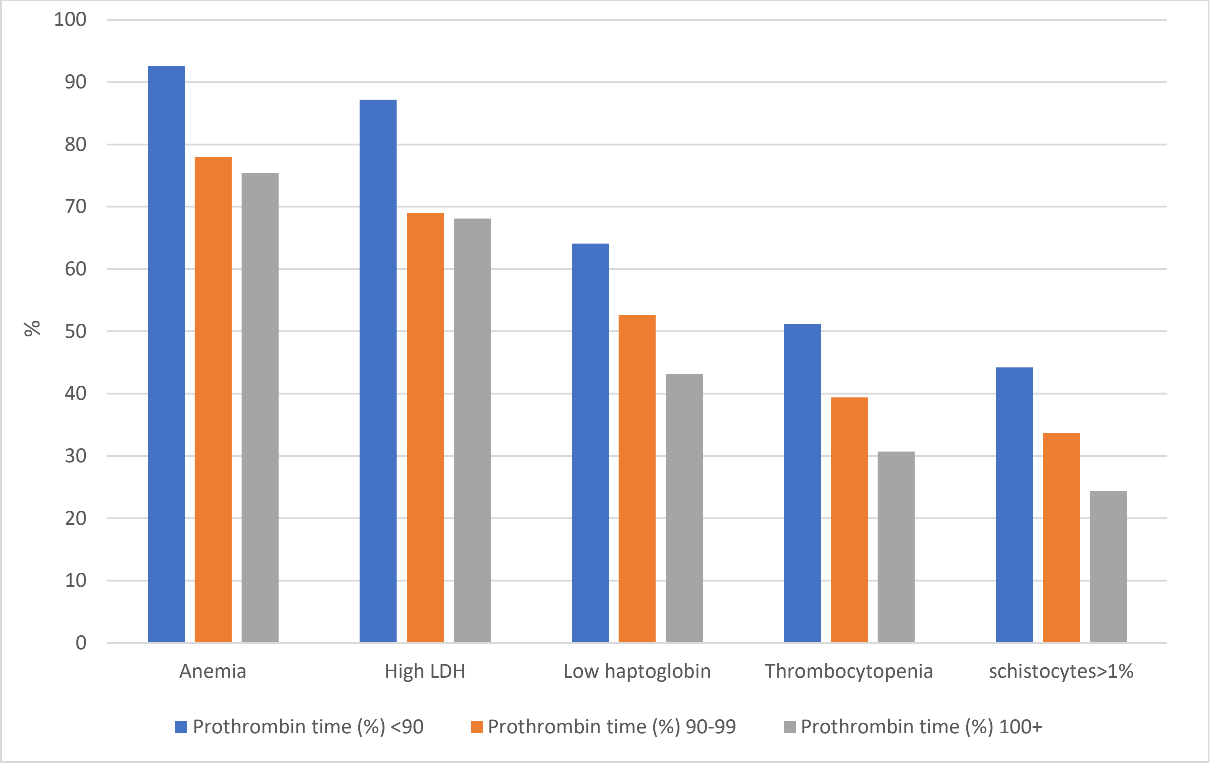

Supplemental Figure 2c: *when the patients were stratified according to serum fibrinogen levels (<4 g/L, 4-4.99 g/L, and ≥5 g/L, the rate of low haptoglobin and schistocytes (but not the rate of anemia, high LDH or thrombocytopenia) decreased in patients with fibrinogen levels ≥ 5 g/L vs the other 2 groups of patients.*

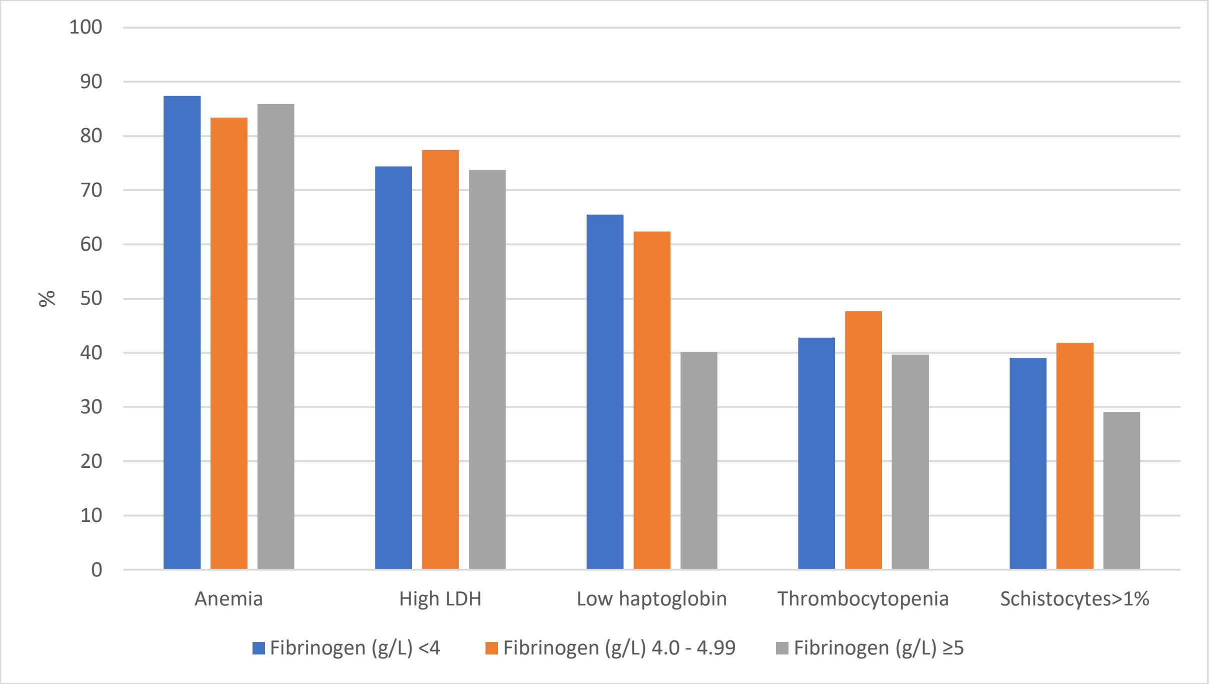

Supplement: Supplementary File (PDF) — Figure S1. Distribution of the 25 MATRIX investigator centers in France. These centers are located throughout metropolitan France. The centers are indicated by a colored circle: red for the coordinating center and green for the other recruiting centers. Figure S2. (A) when patients were separated according to serum creatinine levels (< 200 μmol/l, 200–299 μmol/l and ≥ 300 μmol/l), the rate of all biological parameters of TMA increased with increasing serum creatinine groups from < 200 μmol/l to ≥ 300 μmol/l. (B) When the patients were separated according to prothrombin time (< 90%, 90%–99%, ≥ 100%), the rate of all biological parameters of TMA progressively decreased from < 90% to ≥ 100% prothrombin time groups. (C) When the patients were stratified according to serum fibrinogen levels (< 4 g/l, 4–4.99 g/l, and ≥ 5 g/l), the rate of low haptoglobin and schistocytes (but not the rate of anemia, high LDH, or thrombocytopenia) decreased in patients with fibrinogen levels ≥ 5 g/l versus the other 2 groups of patients. Table S1. Definitions. Table S2. Impact of fibrinogen and serum creatinine on sensitivity of biological parameters to suspect renal TMA. [file mmc1.pdf]
